# Stimulating β-adrenergic receptors promotes synaptic potentiation by switching CaMKII movement from LTD to LTP mode

**DOI:** 10.1016/j.jbc.2023.104706

**Published:** 2023-04-13

**Authors:** Matthew E. Larsen, Olivia R. Buonarati, Hai Qian, Johannes W. Hell, K. Ulrich Bayer

**Affiliations:** 1 Department of Pharmacology, University of Colorado Anschutz Medical Campus, Aurora, Colorado, USA; 2 Program in Neuroscience, University of Colorado Anschutz Medical Campus, Aurora, Colorado, USA; 3 Department of Pharmacology, University of Iowa, Iowa City, Iowa, USA; 4 Department of Pharmacology, University of California at Davis, Davis, California, USA

**Keywords:** synaptic plasticity, long-term potentiation (LTP), long-term depression (LTD), Ca^2+^/calmodulin-dependent protein kinase II (CaMKII), N-methyl-D-aspartate receptor (NMDA receptor, NMDAR), β-adrenergic receptors (βARs), L-type Ca^2+^-channels hippocampus

## Abstract

Learning, memory, and cognition are thought to require synaptic plasticity, specifically including hippocampal long-term potentiation and depression (LTP and LTD). LTP *versus* LTD is induced by high-frequency stimulation *versus* low-frequency, but stimulating β-adrenergic receptors (βARs) enables LTP induction also by low-frequency stimulation (1 Hz) or theta frequencies (∼5 Hz) that do not cause plasticity by themselves. In contrast to high-frequency stimulation-LTP, such βAR-LTP requires Ca^2+^-flux through L-type voltage-gated Ca^2+^-channels, not N-methyl-D-aspartate–type glutamate receptors. Surprisingly, we found that βAR-LTP still required a nonionotropic scaffolding function of the N-methyl-D-aspartate–type glutamate receptor: the stimulus-induced binding of the Ca^2+^/calmodulin-dependent protein kinase II (CaMKII) to its GluN2B subunit that mediates CaMKII movement to excitatory synapses. In hippocampal neurons, β-adrenergic stimulation with isoproterenol (Iso) transformed LTD-type CaMKII movement to LTP-type movement, resulting in CaMKII movement to excitatory instead of inhibitory synapses. Additionally, Iso enabled induction of a major cell-biological feature of LTP in response to LTD stimuli: increased surface expression of GluA1 fused with super-ecliptic pHluorein. Like for βAR-LTP in hippocampal slices, the Iso effects on CaMKII movement and surface expression of GluA1 fused with super-ecliptic pHluorein involved L-type Ca^2+^-channels and specifically required β2-ARs. Taken together, these results indicate that Iso transforms LTD stimuli to LTP signals by switching CaMKII movement and GluN2B binding to LTP mode.

Norepinephrine is famous for its participation in the fight or flight response *via* stimulating β-adrenergic receptors (βARs), but it can also affect memory. On a cellular level, learning and memory are thought to require changes in the number of synaptic AMPA-type glutamate receptors, which can cause long-term changes in synaptic strength such as hippocampal long-term potentiation (LTP) and long-term depression (LTD) (1, 2, 3, 4, 5). Induction of LTP and some forms of LTD typically requires Ca^2+^-influx through N-methyl-D-aspartate (NMDA)-type glutamate receptors (NMDARs) but with distinct stimulation patterns; hippocampal LTP is typically induced by brief high-frequency stimulation (HFS; such as 1-4x 1 s at 100 Hz) that causes brief but strong Ca^2+^-stimuli, whereas LTD is typically induced by low-frequency stimulation (LFS; such as 15 min at 1 Hz) that causes weak but prolonged Ca^2+^-stimuli (6, 7, 8). Interestingly, β-adrenergic stimulation with isoproterenol (Iso) enables LTP induction even after low-frequency stimuli (1–5 Hz for 15 min) that otherwise instead induce LTD (9). Most commonly, such βAR-LTP has been induced with a stimulus that does not induce any plasticity at all on its own: prolonged theta-frequency tetanus of 5 Hz for 3 min (10, 11). This specific form of βAR-LTP has also been named for the type of electrical stimulation, *i.e.*, PTT-LTP, as it mimics naturally occurring brain wave patterns (12, 13). In contrast to HFS-induced LTP, this βAR/PTT-LTP is blocked by L-type Ca^2+^ channel inhibition or by conditional Cav1.2 knockout but is reduced only partially by the glutamate-competitive NMDAR inhibitor APV and is not reduced at all by the NMDAR pore-blocker MK801 that prevents Ca^2+^-influx through NMDARs (13).

We show here that βAR-LTP still requires the NMDAR for its regulated scaffolding of Ca^2+^/calmodulin-dependent protein kinase II (CaMKII) *via* the binding site around S1303 of the NMDAR subunit GluN2B. Thus, even though βAR-LTP is not dependent on ionotropic NMDAR signaling (*i.e.*, it does not require Ca^2+^ flux through NMDARs), it is completely dependent on the nonionotropic scaffolding function of the NMDAR that mediates CaMKII targeting to excitatory synapses. Indeed, we show that β-adrenergic stimulation redirects CaMKII movement from inhibitory to excitatory synapses after LTD stimuli, *i.e.*, the movement normally seen only after LTP stimuli.

## Results

### βAR-LTP requires CaMKII stimulation and its binding GluN2B

**Figure 1:**
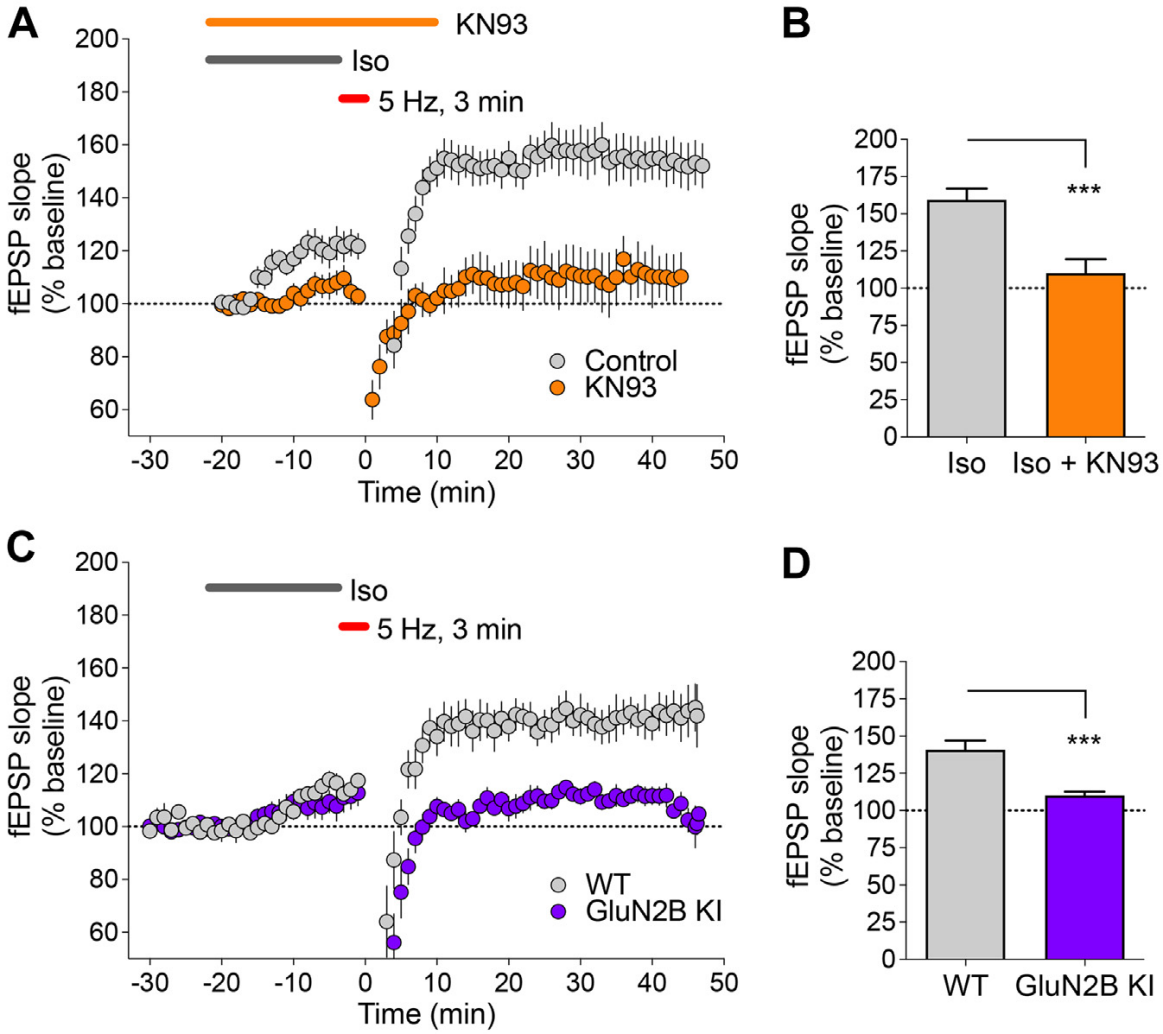
**CaMKII binding to GluN2B is required for βAR-LTP.** Graphs show fEPSP initial slopes recorded from hippocampal CA1 before and after 3 min, 5 Hz stimulation. *Gray bars* indicate perfusion with 1 μM isoproterenol (Iso). Quantifications show mean ± SEM. ∗∗∗*p* < 0.001. *A*, LTP induced by Iso/5 Hz 3 min is blocked by 10 μM KN-93. *B*, potentiation was 159.5 ± 7.4% for Iso and 110.12 ± 9.3% for Iso + KN93 (*p* < 0.001 baseline *versus* Iso, 8 mice, 12 slices; ns baseline *versus* Iso + KN93, 3 mice, four slices). *C*, LTP induced by iso/5 Hz 3 min stimulation requires CaMKII binding to GluN2B, as potentiation is blocked in slices from GluN2B^ΔCaMKII^ KI mice. *D*, potentiation was 140.8 ± 6.2% for slices from wildtype mice and 110.2 ± 2.5% for slices from GluN2B^ΔCaMKII^ KI mice (*p* < 0.001 baseline *versus* wildtype, ns baseline *versus* GluN2B^ΔCaMKII^ KI; 3 mice, 5 slices per genotype). βAR, β-adrenergic receptor; CaMKII, Ca2+/calmodulin-dependent protein kinase II; fEPSP, field excitatory postsynaptic potentials; LTP, long-term potentiation.

In contrast to HFS-induced LTP, the βAR/PTT-LTP that is induced in hippocampal slices by 3 min of 5 Hz stimulation in the presence of 1 μM Iso requires Ca^2+^ influx through the L-type Ca^2+^ channel Cav1.2 but not through the NMDAR (13). However, like HFS-LTP, this βAR-LTP was dependent on CaMKII, as it was completely blocked by the Ca^2+^/CaM-competitive CaMKII inhibitor KN93 (Fig. 1, *A* and *B*). Thus, we decided to additionally test if the βAR-LTP also still requires the CaMKII binding to the NMDAR subunit GluN2B that is necessary for normal HFS-induced LTP (14, 15). For this purpose, we used the GluN2B^ΔCaMKII^ mutant mice (15) that carry two point mutations in GluN2B (L1298A/R1300Q), which together completely disrupt CaMKII binding (16, 17). Notably, the related CaM kinase DAPK1 binds to a partially overlapping site on GluN2B (18, 19), but DAPK1 binding is completely unaffected by the GluN2B^ΔCaMKII^ mutation (17). Remarkably, the GluN2B^ΔCaMKII^ mutation completely blocked the βAR-LTP (Fig. 1, *C* and *D*). Accordingly, even though βAR-LTP does not appear to require the ionotropic NMDAR functions that are necessary for HFS-induced LTP (13), the βAR-LTP is completely dependent on a nonionotropic NMDAR function, providing a scaffold for binding of CaMKII.

### β-adrenergic signaling primes CaMKII for LTP-like synaptic movement upon cLTD stimulation

**Figure 2:**
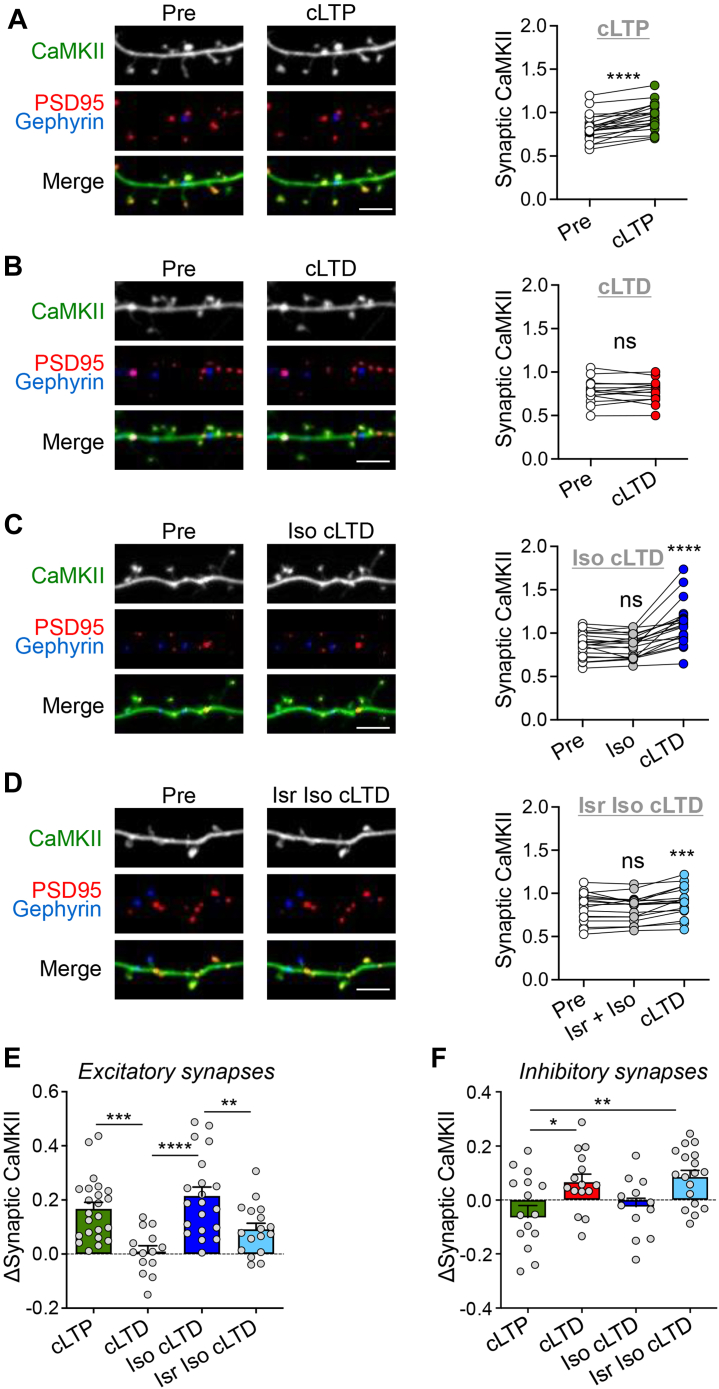
**β-adrenergic signaling primes CaMKII for LTP-like synaptic movement after cLTD stimulation.** Representative confocal images show endogenous CaMKIIα at excitatory synapses (marked by endogenous PSD-95 in *red*) and inhibitory synapses (marked by gephyrin in *blue*) in rat hippocampal neurons (day *in vitro* 16-18). Quantifications show mean ± SEM. ∗*p* < 0.05, ∗∗*p* < 0.01, ∗∗∗*p* < 0.001, ∗∗∗∗*p* < 0.0001, ns indicates no significance. Scale bars, 5 μm. *A*, CaMKII moves to excitatory synapses after cLTP (100 μM glutamate, 10 μM glycine, 1 min) (paired two-tailed *t* test: pre *versus* cLTP ∗∗∗∗*p* < 0.0001, n = 22 cells). *B*, no CaMKII movement to excitatory synapses is observed after cLTD (30 μM NMDA, 10 μM glycine, 10 μM CNQX, 3 min) (paired two-tailed *t* test: pre *versus* cLTD *p* = 0.8348, n = 14 cells). *C*, CaMKII moves to excitatory synapses when cLTD follows pretreatment with isoproterenol (1 μM Iso, 5 min) (repeated-measures one-way ANOVA, Tukey test: pre *versus* Iso *p* = 0.8175, pre *versus* Iso cLTD ∗∗∗∗*p* < 0.0001, Iso *versus* Iso cLTD ∗∗∗∗*p* < 0.0001, n = 20 cells). *D*, CaMKII movement after Iso cLTD is weakened by isradipine (10 μM Isr, 10 min) (repeated-measures one-way ANOVA, Tukey test: pre *versus* Isr Iso *p* = 0.7019, pre *versus* Isr Iso cLTD ∗∗*p* = 0.0036, Isr Iso *versus* Isr Iso cLTD ∗∗∗*p* = 0.0008, n = 17 cells). *E*, quantified change in CaMKII movement to excitatory synapses (one-way ANOVA, Tukey test: cLTP *versus* cLTD ∗∗∗*p* = 0.0007, cLTD *versus* Iso cLTD ∗∗∗∗*p* < 0.0001, Iso cLTD *versus* Isr Iso cLTD ∗∗*p* = 0.0092, cLTP *versus* Isr Iso cLTD *p* = 0.1751, cLTP *versus* Iso cLTD *p* = 0.5321, cLTD *versus* Isr Iso cLTD *p* = 0.2060). *F*, quantified change in CaMKII movement to inhibitory synapses (one-way ANOVA, Tukey test: cLTP *versus* cLTD ∗*p* = 0.0308, cLTP *versus* Isr Iso cLTD ∗∗*p* = 0.0068, cLTP *versus* Iso cLTD *p* = 0.8489, cLTD *versus* Iso cLTD *p* = 0.2675, cLTD *versus* Isr Iso cLTD *p* = 0.9761, Iso cLTD *versus* Isr Iso cLTD *p* = 0.1112). CaMKII, Ca2+/calmodulin-dependent protein kinase II; cLTD, chemical LTD; cLTP, chemical LTP; LTD, long-term depression; LTP, long-term potentiation; NMDA, N-methyl-D-aspartate; NMDAR, NMDA receptor.

The Ca^2+^/CaM-induced CaMKII binding to GluN2B mediates the further accumulation of CaMKII at excitatory synapses within dendritic spines in response to LTP-inducing stimuli (15, 20). By contrast, LTD-inducing stimuli cause CaMKII movement to inhibitory synapses instead (21, 22). As β-adrenergic stimulation with Iso caused LTP induction in response to stimuli that are otherwise more LTD-related, we decided to determine how Iso may modulate the effects of LTD stimuli on synaptic CaMKII movement. For this purpose, we used intrabody-based simultaneous live-imaging of endogenous CaMKII and two marker proteins for excitatory *versus* inhibitory synapses, PSD95 and gephyrin (17, 21). As expected, CaMKII accumulated at excitatory synapses of hippocampal neurons in response to chemical LTP stimuli (cLTP; 1 min 100 μM glutamate/10 μM glycine) (Fig. 2*A*), but not in response to chemical LTD stimuli (cLTD; 3 min 30 μM NMDA, 10 μM glycine, 10 μM CNQX in the presence of Mg^2+^) (Fig. 2*B*). However, pretreatment with Iso (1 μM) enabled CaMKII movement to excitatory synapses also in response to cLTD stimuli (Fig. 2*C*). Similar to the βAR-LTP in hippocampal slices (13), this Iso-enabled redirection of CaMKII movement was suppressed by the L-type Ca^2+^ channel inhibitor isradipine (Isr) (10 μM) (Fig. 2, *D* and *E*); even though some residual movement to excitatory synapses was still observed (Fig. 2*D*), the CaMKII movement was significantly reduced by the addition of Isr (Fig. 2*E*).

### β-adrenergic stimulation blocks CaMKII movement to inhibitory synapses upon cLTD stimulation

The reciprocal effect was observed for CaMKII movement to inhibitory synapses, which was significantly greater in response to cLTD-inducing *versus* cLTP-inducing stimuli (Fig. 2*F*), as expected based on previous studies (21, 23). Adding β-adrenergic stimulation with 1 μM Iso before the cLTD stimulus blocked any movement to inhibitory synapses, but additional inhibition of L-type Ca^2+^ channels with Isr restored the movement to the same degree as seen after cLTD without β-adrenergic stimulation (Fig. 2*F*). These data indicate that β-adrenergic stimulation can transform LTD-type stimuli to LTP signals by redirecting CaMKII movement to excitatory synapses. Similar to βAR-LTP in hippocampal slices, the β-adrenergic effect on LTP-like CaMKII movement required L-type Ca^2+^ channels.

### Iso-induced CaMKII redirection to excitatory synapses requires GluN2B binding

**Figure 3:**
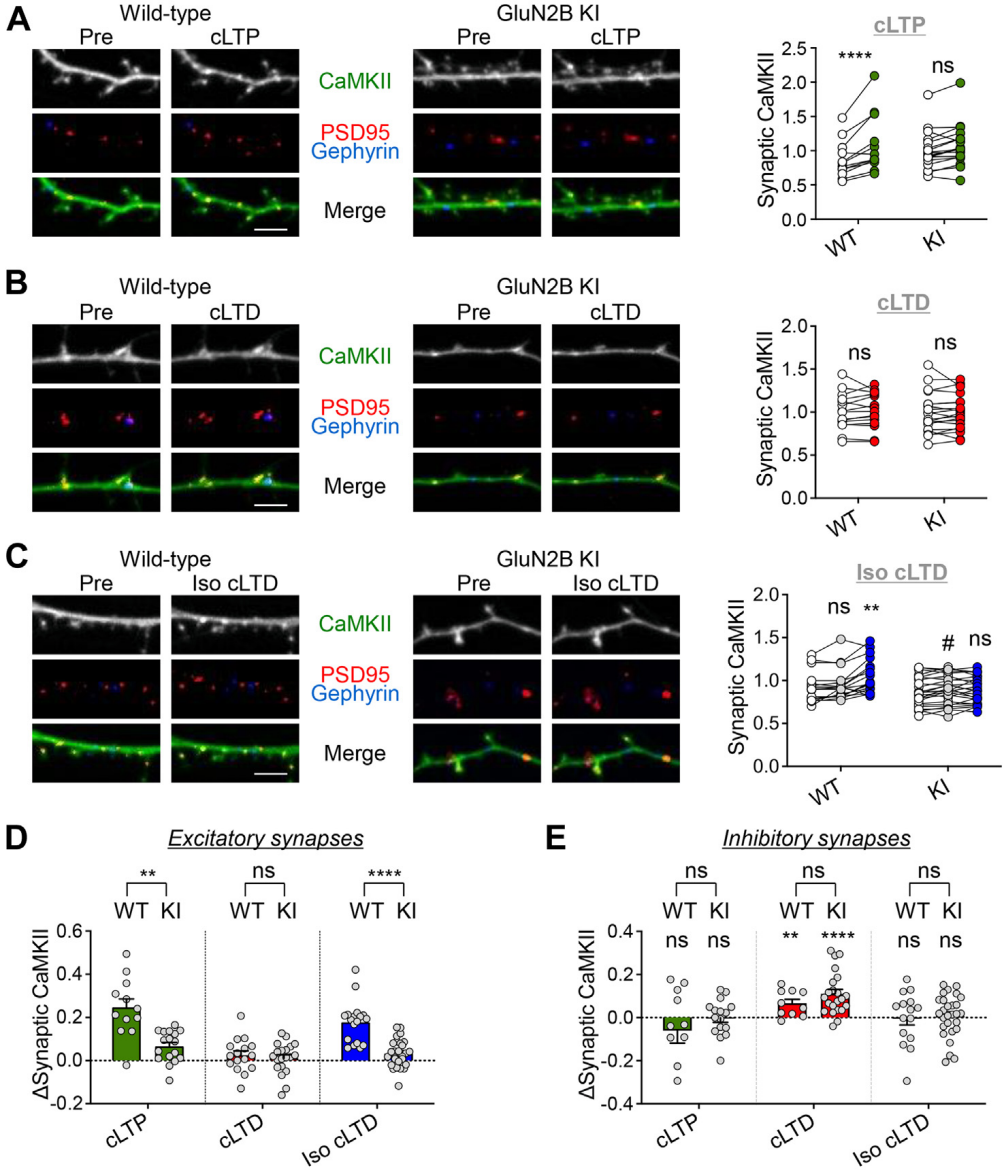
**Glu2N2B binding is required for the β-adrenergic switch in synaptic CaMKII movement.** Representative confocal images show endogenous CaMKIIα at excitatory synapses (marked by endogenous PSD-95 in *red*) and inhibitory synapses (marked by gephyrin in *blue*) in hippocampal neurons (day *in vitro* 16-18) cultured from wildtype *versus* GluN2B^ΔCaMKII^ mice. Quantifications show mean ± SEM. ∗*p* < 0.05, ∗∗*p* < 0.01, ∗∗∗*p* < 0.001, ∗∗∗∗*p* < 0.0001, ns indicates no significance. Scale bars, 5 μm. *A*, CaMKII moves to excitatory synapses after cLTP (100 μM glutamate, 10 μM glycine, 1 min) in WT but not GluN2B^ΔCaMKII^ KI neurons (two-way ANOVA, Bonferroni’s test: WT pre *versus* cLTP ∗∗∗∗*p* < 0.0001, n = 12 cells; GluN2B KI pre *versus* cLTP *p* = 0.0528, n = 19). *B*, no CaMKII movement to excitatory synapses is observed after cLTD (30 μM NMDA, 10 μM glycine, 10 μM CNQX, 3 min) in either WT or KI (two-way ANOVA, Bonferroni’s test: WT pre *versus* cLTD *p* > 0.9999, n = 15 cells; GluN2B KI pre *versus* cLTD *p* > 0.9999, n = 20 cells). *C*, CaMKII moves to excitatory synapses when cLTD is pretreated with isoproterenol (1 μM Iso, 5 min) only in WT (two-way ANOVA, Bonferroni’s test: pre *versus* Iso *p* = 0.1301, pre *versus* Iso cLTD ∗∗∗∗*p* < 0.0001, Iso *versus* Iso cLTD ∗∗*p* = 0.0028, n = 18 cells). Iso cLTD-induced movement to excitatory synapses is blocked in KI (two-way ANOVA, Bonferroni’s test: pre *versus* Iso #*p* = 0.0427, pre *versus* Iso cLTD *p* = 0.0839, Iso *versus* Iso cLTD *p* = 0.9968, n = 25 cells). *D*, quantified change in CaMKII movement to excitatory synapses. GluN2B^ΔCaMKII^ KI impairs CaMKII synaptic enrichment compared to WT (two-way ANOVA, Bonferroni’s test: WT *versus* KI cLTP ∗∗*p* = 0.0025, cLTD *p* > 0.9999, Iso cLTD ∗∗∗∗*p* < 0.0001). *E*, quantified change in CaMKII movement to inhibitory synapses is not significantly different between WT and GluN2B^ΔCaMKII^ KI (two-way ANOVA, Bonferroni’s test: WT *versus* KI cLTP *p* > 0.9999, cLTD *p* = 0.4009, Iso cLTD *p* > 0.9999). CaMKII, Ca2+/calmodulin-dependent protein kinase II; cLTD, chemical LTD; cLTP, chemical LTP; NMDA, N-methyl-D-aspartate; NMDAR, NMDA receptor.

The activity-induced accumulation of CaMKII at excitatory synapses in response to LTP-stimuli requires Ca^2+^/CaM-induced binding to GluN2B (15, 20). Thus, we decided to test if this is also the case for the redirection of CaMKII movement to dendritic spines that was seen here after β-adrenergic stimulation with Iso. For this purpose, we compared CaMKII movement in neurons from either wildtype mice or GluN2B^ΔCaMKII^ mice that have a GluN2B mutation that is CaMKII binding-incompetent (16, 17). Compared to rat cultures, the mouse cultures appeared to have lower spine densities, but this was the same for both genotypes, consistent with previous studies on cultures from the GluN2B^ΔCaMKII^ mice (15). As expected, CaMKII moved to excitatory synapses in response to cLTP stimuli in hippocampal neurons from mouse, and this movement was blocked by the GluN2B^ΔCaMKII^ mutation (Fig. 3*A*). As also expected, cLTD stimuli did not cause any CaMKII movement to excitatory synapses, neither in wildtype nor in GluN2B^ΔCaMKII^ neurons (Fig. 3*B*). However, β-adrenergic stimulation with Iso enabled CaMKII movement in response cLTD stimuli and, as with cLTP stimuli, this movement was abolished by the GluN2B^ΔCaMKII^ mutation (Fig. 3, *C* and *D*). Thus, CaMKII binding to GluN2B is required for both (i) the Iso-induced redirection of synaptic CaMKII movement and (ii) the βAR-LTP that is otherwise independent of NMDAR current.

### The GluN2B^ΔCaMKII^ mutation did not cause CaMKII redirection to inhibitory synapses

Whereas the GluN2B^ΔCaMKII^ mutation abolished the LTP-related CaMKII movement to excitatory synapses (Fig. 3*D*), it did not affect CaMKII movement to inhibitory synapses at all (Fig. 3*E*); it neither prevented the LTD-related movement to inhibitory synapses nor induced such movement in response to LTP-related stimuli. This finding is consistent with the fact that in contrast to Iso or Isr, the GluN2B^ΔCaMKII^ mutation does not change the nature of the upstream activation of CaMKII but only abolishes CaMKII localization at excitatory synapses, which should not affect localization to inhibitory synapses.

### β-adrenergic stimulation enabled SEP-GluA1 surface insertion in response to cLTD stimuli

**Figure 4:**
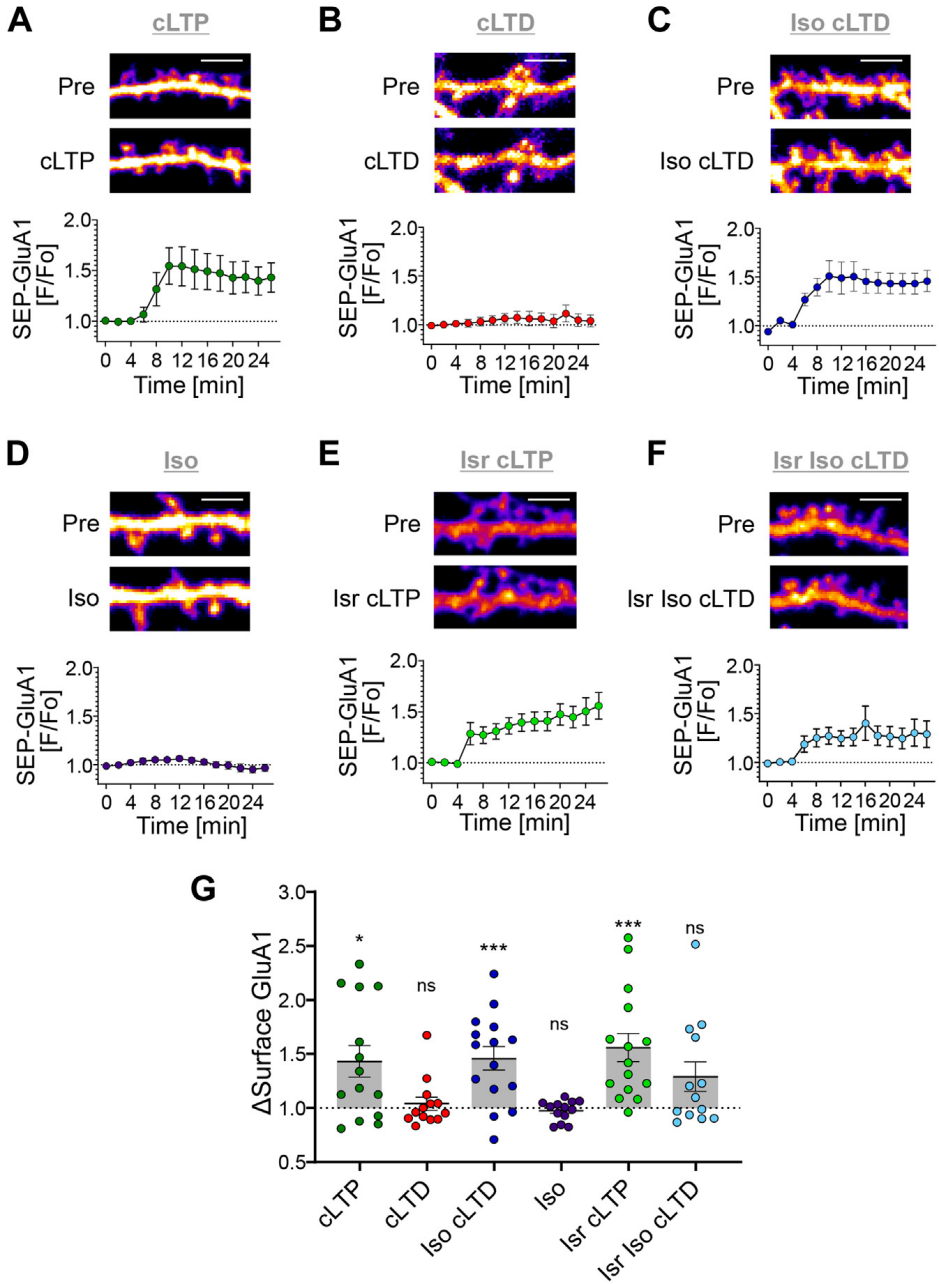
**β-adrenergic stimuli allow GluA1 surface insertion in response to LTD stimuli.** Representative confocal images show SEP-GluA1 in hippocampal neurons (day *in vitro* 16-18) cultured from rats. Quantifications show mean ± SEM. ∗*p* < 0.05, ∗∗∗*p* < 0.001, ns indicates no significance. Scale bars, 5 μm. *A*, cLTP stimuli (100 μM glutamate, 10 μM glycine, 1 min) induced SEP-GluA1 surface insertion. *B*, cLTD (30 μM NMDA, 10 μM glycine, 10 μM CNQX, 3 min) stimuli do not induce SEP-GluA1 surface insertion. *C*, in the presence of β-adrenergic stimulation with isoproterenol (1 μM Iso, 5 min), SEP-GluA1 surface insertion was triggered even by cLTD stimuli. *D*, on its own, 1 μM Iso had no effect on SEP-GluA1 surface expression. *E*, inhibition of L-type Ca^2+^ channels with 10 μM isradipine (Isr) did not affect the SEP-GluA1 insertion induced by cLTP. *F*, by contrast, inhibition of L-type Ca^2+^ channels with Isr reduced SEP-GluA1 insertion induced by Iso cLTD, and the remaining apparent insertion was no longer statistically significant. *G*, combined bar graphs. Inhibition of L-type Ca^2+^ channels with Isr (10 μM Isr, 10 min) did not interfere with the SEP-GluA1 insertion that is induced by cLTP; however, the insertion in response to iso + cLTD stimuli was no longer significant (one-sample *t* test: cLTP ∗*p* = 0.0111, cLTD ns *p* = 0.545, Iso cLTD ∗∗∗*p* = 0.0008, Iso ns *p* = 0.3549, Isr cLTP ∗∗∗*p* = 0.008, Isr Iso cLTD ns *p* = 0.056). cLTD, chemical LTD; cLTP, chemical LTP; LTD, long-term depression; NMDA, N-methyl-D-aspartate; NMDAR, NMDA receptor; SEP-GluA1, GluA1 fused with super-ecliptic pHluorein.

Our results showed that β-adrenergic stimulation redirects CaMKII to an LTP-like movement in response to cLTD stimuli in hippocampal neurons. This movement was mediated by GluN2B binding at excitatory synapses, which was required for the βAR-LTP in hippocampal slices. However, in contrast to the βAR-LTP in hippocampal slices, our iso+cLTD stimuli in hippocampal neurons strictly depend on NMDAR signaling, as the cLTD stimulus with 30 μM NMDAR acts by directly activating the NMDAR, without direct activation of other glutamate receptors. Thus, we decided to test the effect of the iso+cLTD stimulus also on increasing the surface expression of the AMPA-type glutamate receptor subunit GluA1, the hallmark feature of LTP induction. GluA1 surface insertion was measured by expressing GluA1 fused with super-ecliptic pHluorein (SEP-GluA1), *i.e.*, a GluA1 fusion protein with super-ecliptic fluorein, which has quenched fluorescence at the low pH found in vesicles and then increases in fluorescence upon surface insertion (24). As expected, SEP-GluA1 surface insertion increased significantly only after cLTP but not after cLTD stimuli (Fig. 4, *A* and *B*). However, in presence of 1 μM Iso, even cLTD stimuli triggered an increase in SEP-GluA1 surface (Fig. 4*C*). Without other stimuli, Iso had no effect on SEP-GluA1 surface expression in our neuronal cultures (Fig. 4*D*) (but see (25). Like the βAR-LTP in hippocampal slices and the CaMKII movement in hippocampal neurons, this iso+cLTD-induced SEP-GluA1 insertion in hippocampal neurons was sensitive to inhibition of L-type Ca^2+^ channels: while cLTP-induced SEP-GluA1 surface insertion was not affected by 10 μM Isr (Fig. 4*E*), the SEP-GluA1 insertion induced by cLTP stimuli in presence of Iso was at least partially sensitive to such inhibition of L-type Ca^2+^ channels (Fig. 4, *F* and *G*). Thus, β-adrenergic stimulation of hippocampal neurons with Iso switches both CaMKII movement and GluA1 surface insertion from LTD to LTP mode.

### The Iso-induced switch of CaMKII movement depends on the β2-AR

**Figure 5:**
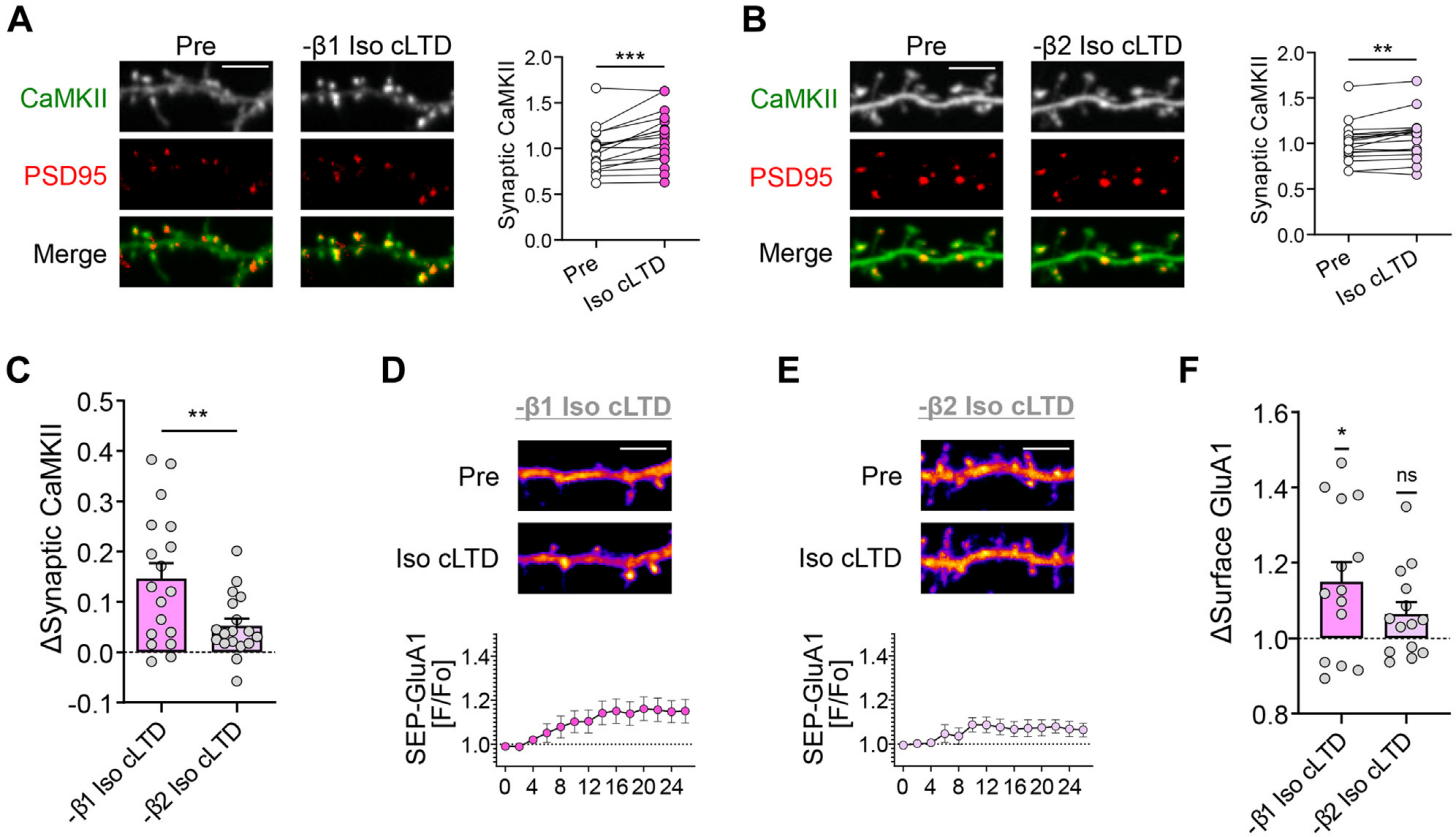
**β2-adrenergic stimuli allow CaMKII movement and GluA1 surface insertion in response to LTD stimuli.** Representative confocal images show endogenous CaMKII at excitatory synapses (marked by endogenous PSD-95 in *red*) or SEP-GluA1 in hippocampal neurons (day *in vitro* 16-18) cultured from rats. Quantifications show mean ± SEM. ∗*p* < 0.05, ∗∗*p* < 0.01, ∗∗∗*p* < 0.001, ns indicates no significance. Scale bars, 5 μm. *A*, CaMKII moves to excitatory synapses following isoproterenol treatment (1 μM Iso, 5 min) and cLTD (30 μM NMDA, 10 μM glycine, 10 μM CNQX, 3 min) even after pretreatment with a β1-AR antagonist (500 nM CGP-20712, 15 min) (paired *t* test: ∗∗∗*p* = 0.0005), to the same extent as seen without antagonist (compare to Fig. 2*C*; unpaired *t* test: *p* = 0.1393). *B*, following pretreatment with a β2-AR antagonist (200 nM ICI-118,551, 15 min), some residual movement of CaMKII to excitatory synapses was still detected in response to Iso cLTD (paired *t* test: ∗∗*p* = 0.0014); however, this movement was not statistically different from cLTD conditions without any βAR stimulation (compare to Fig. 2*B*; unpaired *t* test: *p* = 0.0941). *C*, the movement observed in presence of β2-AR inhibition was significantly reduced compared to the movement in presence of β1-AR inhibition (unpaired *t* test: ∗∗*p* = 0.0086). *D*, Iso cLTD induced SEP-GluA1 surface insertion following pretreatment with a β1-AR antagonist. *E*, Iso cLTD failed to induce SEP-GluA1 insertion following pretreatment with a β2-AR antagonist. *F*, quantification shows significant insertion of SEP-GluA1 in presence of the β1-AR antagonist but not in presence of the β2-AR antagonist (one-sample *t* test: -β1 Iso cLTD ∗*p* = 0.0133, -β2 Iso cLTD ns *p* = 0.0623). βAR, β-adrenergic receptor; CaMKII, Ca2+/calmodulin-dependent protein kinase II; cLTD, chemical LTD; LTD, long-term depression; NMDA, N-methyl-D-aspartate; NMDAR, NMDA receptor; SEP-GluA1, GluA1 fused with super-ecliptic pHluorein.

Finally, since the βAR/PTT-LTP that is enabled by Iso in hippocampal slices depends on β2-ARs rather than β1-ARs (26), we decided to test if this is also the case for the Iso-enabled switch of the mode of synaptic CaMKII movement. Indeed, the LTP-type movement of CaMKII to excitatory synapses in response to cLTD stimuli in hippocampal neurons in the presence of Iso was not affected by the β1-AR antagonist CGP-20712 (500 nM) (Fig. 5*A*; compared to movement without inhibitor in Fig. 2*C*). In presence of the β2-AR antagonist ICI-118,551 (200 nM), some minimal CaMKII movement to excitatory synapses was still detected in response to cLTD stimuli in presence of Iso (Fig. 5*B*), but this movement was significantly reduced compared to the β1-AR inhibition (Fig. 5*C*), indicating that the Iso effect on CaMKII movement is mediated at least for the most part by β2-ARs. Similarly, the iso+cLTD-induced SEP-GluA1 insertion in hippocampal neurons was blocked by the inhibition of β2-ARs but not by inhibition of β1-ARs (Fig. 5, *D*–*F*), further mechanistically linking βAR-LTP to the βAR-mediated redirection of synaptic CaMKII movement.

## Discussion

Combining electrical 5 Hz stimulation with β-adrenergic stimulation by Iso enables induction of βAR-LTP (also termed PTT-LTP) that depends on L-type Ca^2+^ channels but not on Ca^2+^ influx through NMDARs (13). However, this study shows that βAR-LTP still requires a structural, nonionotropic NMDAR function: synaptic targeting of CaMKII *via* binding to the NMDAR subunit GluN2B. This binding was absolutely required for βAR-LTP and was responsible for the Iso-induced transformation of activity-induced CaMKII accumulation at excitatory synapses from LTD mode to LTP mode. Notably, βAR-LTP appeared to be even more dependent on CaMKII/GluN2B binding than the HFS-LTP that requires NMDR receptors also for Ca^2+^-influx: genetic disruption of CaMKII binding to GluN2B reduced HFS-LTP by approximately half (15) but abolished βAR-LTP almost completely. This finding also indicates that the most important LTP function of CaMKII binding to GluN2B is not sensitization to local NMDAR Ca^2+^ signals but rather facilitation of downstream signaling, such as positioning near effector substrates.

The apparent stronger requirement for CaMKII/GluN2B binding in βAR-LTP compared to the “traditional” HFS-LTP could be due to partial compensation in the GluN2B^ΔCaMKII^ mice in which this binding is chronically disabled. Indeed, more acute interventions with CaMKII/GluN2B binding appear to have a stronger effect on such LTP mechanisms (27, 28). Such compensatory mechanisms for only “traditional” LTP but not βAR-LTP are also consistent with the behavioral findings in the GluN2B^ΔCaMKII^ mice: the modest impairments during the memory consolidation phase in the Morris water maze seen previously (15) match the corresponding memory impairment seen when endogenous β-adrenergic signaling with norepinephrine is prevented (29). These modest behavioral effects would be expected from specific impairment of βAR-LTP, whereas general LTP impairment would be expected to lead to a more severe impairment of learning and memory.

HFS-LTP is thought to undergo a developmental switch from PKA to CaMKII dependence (30). By contrast, both kinases appear to be corequired for the βAR-LTP even in adulthood: requirement for phosphorylation of PKA sites on L-type channels in response to β-adrenergic stimulation has been shown previously (13, 31) and our data demonstrate an additional requirement of CaMKII. Both kinases clearly depend on highly localized signaling in order to enable the β-adrenergic conversion of low frequency stimulation to LTP: PKA requires anchoring by AKAP79/150 (32) and CaMKII by GluN2B (shown here). This may provide important mechanisms for how norepinephrine can act as a molecular switch to facilitate acuity during novel, emotionally charged situations (which would otherwise be less memorable).

Notably, for hippocampal LTD, nonionotropic functions of the NMDAR have been described to require NMDAR signaling, but not Ca^2+^-flux through the receptor (33, 34, 35). However, this finding remains somewhat controversial (35), as others have found block of LTD not only by glutamate-competitive inhibitors but also by the pore blocker MK801 (7, 36, 37, 38, 39, 40). Interestingly, a requirement of CaMKII for LTD has been described not only in studies that found the NMDAR function to be ionotropic (40) but also in studies that described nonionotropic NMDAR functions instead (41, 42). However, the CaMKII binding to GluN2B is a nonionotropic NMDAR function that is specific for LTP: not only is LTD normal in the GluN2B^ΔCaMKII^ mutant mice (15) but also LTD even requires specific suppression of the CaMKII binding to GluN2B (19, 23). Also, the nonionotropic NMDAR function in LTD still requires NMDAR signaling induced by glutamate binding. Involvement of glutamate binding is less clear for the nonionotropic scaffolding function in LTP: even though glutamate-competitive NMDAR inhibitors did not completely block βAR-LTP, they still showed some reduction of βAR-LTP, whereas the channel blocker MK801 had no effect on βAR-LTP in slices at all (13). The mechanisms that determine if CaMKII induces LTP or LTD await further elucidation. However, the current study clearly demonstrates that the direction of the LTP *versus* LTD decision by CaMKII is strongly modulated by β-adrenergic signals, both in hippocampal slices and in cultured hippocampal neurons. As previously observed for βAR-LTP in slices (26), the effect on CaMKII movement in hippocampal neurons was specifically dependent on β2-AR. While βAR-LTP is typically experimentally induced in the presence of Iso, a similar effect is also elicited by endogenous ligands such as norepinephrine (43), indicating that the effect of β2-AR stimulation on enabling LTP can be elicited also in presence of additional stimulation of both β1-ARs and αARs.

In earlier work, we found that Iso by itself can stimulate surface insertion of endogenous GluA1 and of ectopically SEP-GluA1 when Iso was applied into the culture medium that contains the B27 or related NS21 supplement (25). In the current work, we replaced the culture medium with artificial cerebral spinal fluid (ACSF) before application of Iso, which clearly prevented Iso by itself to directly induce surface insertion of GluA1. Apparently, the culture medium, which has a complex composition (44), contains one or more factors that are not only important for the health of the neurons in culture but also promote GluA1 surface expression so that Iso alone can acutely stimulate it. Defining such factors will be an interesting question for the future, which is now possible to pursue based on our finding of this difference between culture medium and ACSF.

## Experimental procedures

### Material availability

Requests for resources, reagents, or questions about methods should be directed to K. Ulrich Bayer (ulli.bayer@cuanschutz.edu). This study did not generate new unique reagents.

### Animal and cell culture models

All animal procedures were approved by the Institutional Animal Care and Use Committee of the University of Colorado Anschutz Medical Campus or of the University of California at Davis; all procedures were carried out in accordance with NIH best practices for animal use. All animals were housed in ventilated cages on a 12 h light/12 h dark cycle and were provided ad libitum access to food and water. Mixed sex WT or mutant mouse littermates (on a C57BL/6 background) from heterozygous breeder pairs were used for slice electrophysiology and biochemistry. Mixed sex pups from homozygous mice (P1-2) or Sprague-Dawley rats (P0) were used to prepare dissociated hippocampal cultures for imaging and biochemistry. GluN2B^ΔCaMKII^ knock-in mutant mice were described previously (15, 19).

### Material and DNA constructs

Material was obtained from Sigma, unless noted otherwise. The expression vectors for the GFP-labeled FingR intrabodies targeting CaMKIIα, PSD-95, and gephyrin were kindly provided by Dr Donald Arnold (University of Southern California) as previously characterized (45, 46). As we have described recently (21), the fluorophore label was exchanged using Gibson Assembly to contain the following tags in place of GFP: CaMKIIα-FingR-YFP2, PSD-95-FingR-mTurquois, and gephyrin-FingR-mCherry.

### Hippocampal slice preparation

WT and mutant mouse hippocampal slices were prepared using adult mice (8–16 weeks old). Isoflurane anesthetized mice were rapidly decapitated, and the brain was dissected in ice-cold high sucrose solution containing (in mM) 220 sucrose, 12 MgSO_4_, 10 glucose, 0.2 CaCl_2_, 0.5 KCl, 0.65 NaH_2_PO_4_, 13 NaHCO_3_, and 1.8 ascorbate. Transverse hippocampal slices (350 μm) were made using a vibratome (Leica VT 1000A) and transferred into 32 °C ACSF containing (in mM) 124 NaCl, 2 KCl, 1.3 NaH_2_ PO4, 26 NaHCO_3_, 10 glucose, 2 CaCl_2_, 1 MgSO_4_, and 1.8 ascorbate. All solutions were recovered in 95% O_2_/5% CO_2_ for at least 1 h before experimentation.

### Extracellular field recordings

All recordings and analysis were performed blind to genotype. For electrical slice recording experiments, a glass micropipette (typical resistance 0.4–0.8 MΩ when filled with ACSF) was used to record field excitatory postsynaptic potentials from the hippocampal CA1 dendritic layer in response to stimulation in the Schaffer collaterals at the CA2 to CA1 interface using a tungsten bi-polar electrode. Slices were continually perfused with 30 °C ACSF at a rate of 2 ml/min during recordings. Stimuli were delivered every 15 s and amplified with an Axopatch 2B amplifier, digitized with a Digidata 1320A, and recorded with Clampex 9 (Molecular Devices). Data were analyzed using WinLTP software (47) with slope calculated as the initial rise from 10 to 60% of response peak. Input/output (I/O) curves were generated by increasing the stimulus intensity at a constant interval until a maximum response or population spike was noted to determine stimulation that elicits 40 to 70% of maximum slope. Slope of I/O curve was calculated by dividing the slope of response (mV/ms) by the fiber volley amplitude (mV) for the initial linear increase. Paired-pulse recordings (50 ms interpulse interval) were acquired from 40% max slope, and no differences in presynaptic facilitation were seen in mutant slices. Iso LTP was induced by bath application of 1 μM Iso, followed by a train of pulses of a frequency of 5 Hz lasting 3 min. The level of LTP was determined by the average field excitatory postsynaptic potential initial slope from the 30 min period between 15 and 45 min after the tetanus.

### Hippocampal culture preparation

To prepare primary hippocampal neurons from WT or mutant mice, hippocampi were dissected from mixed sex mouse pups (P1-2), dissociated in papain, and plated at 200 to 300,000 cells/ml for imaging or 500,000 cells/ml for biochemistry. To prepare rat neurons, hippocampi were dissected from mixed set rat pups (P0), dissociated in papain for 1 h, and plated at 100,000 cells/ml for imaging. At day *in vitro* 14 to 16, neurons were transfected with 1 μg total cDNA per well using Lipofectamine 2000 (Invitrogen), then imaged or treated and fixed 2 to 3 days later.

### cLTD and LTP stimulation

cLTP was induced with 100 μM glutamate and 10 μM glycine for 1 min. cLTD was induced with 30 μM NMDA, 10 μM glycine, and 10 μM CNQX for 3 min. Iso cLTD was induced by pretreating cLTD with 1 μM Iso for 5 min 10 μM Isr was added directly before the Iso pretreatment. 500 nM CGP-20712 (β1-AR antagonist) or 200 nM ICI-118,551 (β2-AR antagonist) was added 15 min prior to the Iso pretreatment. Treatments to induce cLTP or cLTD were followed by washout in fresh ACSF.

### Live imaging of CaMKII movement in hippocampal cultured neurons

All images were acquired using an Axio Observer microscope (Carl Zeiss) fitted with a 63× Plan-Apo/1.4 numerical aperture objective, using 445, 515, 561, and 647 nm laser excitation and a CSU-XI spinning disk confocal scan head (Yokogawa) coupled to an Evolve 512 EM-CCD camera (Photometrics). Experiments were analyzed using Slidebook 6.0 software (Intelligent Imaging Innovations [3i]). During image acquisition, neurons were maintained at 34 °C in ACSF solution containing (in mM): 130 NaCl, 5 KCl, 10 Hepes pH 7.4, 20 glucose, 2 CaCl_2_, and 1 MgCl_2_, adjusted to proper osmolarity with sucrose. After baseline imaging and cLTP or cLTD treatment, neurons were imaged 5 min after washout. Tertiary dendrites from pyramidal spiny neurons were selected from maximum intensity projections of confocal Z stacks. To analyze synaptic CaMKIIα, the mean yellow fluorescent protein (YFP) intensity (CaMKIIα) at excitatory (PSD-95) and inhibitory (gephyrin) synapses was quantified. PSD-95 and gephyrin threshold masks were defined using the mean intensity of mTurquois or mCherry plus two standard deviations. Synaptic CaMKIIα was then calculated using the mean YFP intensity at PSD-95 or gephyrin puncta masks divided by the mean intensity of a line drawn in the dendritic shaft. Changes in CaMKIIα synaptic accumulation were determined by dividing the net change in YFP at PSD-95 or gephyrin puncta-to-shaft ratio by the prestimulation YFP puncta-to-shaft ratio.

### Live imaging of SEP-GluA1 surface expression in hippocampal cultured neurons

All images were acquired using an Axio Observer microscope (Carl Zeiss) fitted with a 63× Plan-Apo/1.4 numerical aperture objective, using 488 and 561 nm laser excitation and a CSU-XI spinning disk confocal scan head (Yokogawa) coupled to an Evolve 512 EM-CCD camera (Photometrics). Experiments were analyzed using Slidebook 6.0 software (Intelligent Imaging Innovations [3i]). During image acquisition, neurons were maintained at 34 °C in ACSF solution containing (in mM): 130 NaCl, 5 KCl, 10 Hepes pH 7.4, 20 glucose, 2 CaCl_2_, and 1 MgCl_2_, adjusted to proper osmolarity with sucrose. After baseline imaging and cLTP and cLTD treatment, neurons were imaged once every 2 min for 20 min after washout. Tertiary dendrites from pyramidal spiny neurons were selected from maximum intensity projections of confocal Z stacks. To analyze surface GluA1 expression, the mean SEP intensity (GluA1) at distinct SEP puncta was quantified. Changes in GluA1 surface expression were determined by dividing the mean SEP intensity in distinct puncta at 20 min after washout by the prestimulation mean SEP intensity.

### Quantification and statistical analysis

All data are shown as mean ± SEM. Statistical significance and sample size (n) are indicated in the figure legends; all imaging experiments were typically conducted in three and at least in two independent cultures for each condition. Data from the imaging experiments were obtained and quantified using SlideBook 6.0 software (3i) and analyzed using Prism (GraphPad) software. To test for parametric conditions, data were evaluated by a Shapiro–Wilk test for normal distribution and a Brown-Forsythe test (three or more groups) or an F-test (two groups) to determine equal variance. Comparisons between two groups were analyzed using unpaired, two-tailed Student’s *t* tests. Comparisons between pretreatment and posttreatment images at the same synapse type from the same neurons were analyzed using paired, two-tailed Student’s *t* tests. Comparisons between three or more groups were done by one-way ANOVA with Tukey’s post-hoc test. Treatment of the same neuron over time was analyzed as repeated-measures. Comparisons between three or more groups with two independent variables were assessed by two-way ANOVA with Bonferroni post-hoc test to determine whether there is an interaction and/or main effect between the variables.

## Data availability

The datasets generated during this study are available through Mendeley. No original code was generated during this study.

## Conflict of interest

K. U. B. is co-founder and board member of Neurexis Therapeutics.
